# Alpha-Theta Effects Associated with Ageing during the Stroop Test

**DOI:** 10.1371/journal.pone.0095657

**Published:** 2014-05-27

**Authors:** Cristina Nombela, Manuel Nombela, Pedro Castell, Teodoro García, Juan López-Coronado, María Trinidad Herrero

**Affiliations:** 1 Clinical and Experimental Neuroscience (NiCE-CIBERNED), School of Medicine, University of Murcia, Murcia, Spain; 2 Clinical Neuroscience Department, University of Cambridge, Cambridge, United Kingdom; 3 Neurophysiology Department, Nuestra Sra. del Rosell Hospital, Murcia, Spain; 4 Department of System Engineering and Automation, Technical University of Cartagena, Murcia, Spain; 5 Clinical and Experimental Neuroscience (NiCE-CIBERNED), School of Health Sciences (Medicine), University Jaume I, Castellón, Spain; Hospital Nacional de Parapléjicos - SESCAM, Spain

## Abstract

The Stroop effect is considered as a standard attentional measure to study conflict resolution in humans. The response of the brain to conflict is supposed to change over time and it is impaired in certain pathological conditions. Neuropsychological Stroop test measures have been complemented with electroencephalography (EEG) techniques to evaluate the mechanisms in the brain that underlie conflict resolution from the age of 20 to 70. To study the changes in EEG activity during life, we recruited a large sample of healthy subjects of different ages that included 90 healthy individuals, divided by age into decade intervals, which performed the Stroop test while recording a 14 channel EEG. The results highlighted an interaction between age and stimulus that was focused on the prefrontal (Alpha and Theta band) and Occipital (Alpha band) areas. We concluded that behavioural Stroop interference is directly influenced by opposing Alpha and Theta activity and evolves across the decades of life.

## Introduction

The classical Stroop test [1] is an executive task used to evaluate prefrontal function that can be applied during the life span of healthy individuals and in neurological pathologies, such as Parkinson's disease [2], Alzheimer's disease [3] and schizophrenia [4]. The test involves the presentation of a series of words (colour names) written in different coloured inks. The ink colour (chromatic information) and the colour's name (semantic information) may be the same (congruent target) or different (incongruent target), demanding the resolution of a cognitive conflict. Accordingly, the subject must respond in function of the ink colour and not the word meaning or the semantic information, and overcoming the automatic response of reading the word produces a delay in the response known as the Stroop interference or Stroop effect.

Some studies suggested that this interference may already occur at the stimulus processing stage [5], an hypothesis that can be verified by measuring evoked response potentials (ERPs) by electroencephalography (EEG), comparing the intensity and signal delays between congruent and incongruent targets [6]. EEG recordings showed that incongruent stimuli have no effect on the amplitude or latency of the P300 component -the cognitive evoked potential-[7], although they induce stronger negativity at around 400 ms than neutral stimuli [8]. This would suggest that interference analysis occurs quite late in time, closer to the response stage than to the stimulus processing stage.

The specific nature of the Stroop effect can also be studied by instantaneous coherence analysis based on a Fast Fourier Transformation (FFT) and in relation to band frequency studies. It was proposed that the 13–20 Hz frequency band was sensitive to discrimination between the congruent and incongruent items, and that higher coherence was observed within the left frontal and left parietal areas [9]–[11]. This is consistent with more recent findings regarding coherence within a time interval of 100–400 ms at 13–18 Hz, which was higher for incongruent situations than for congruent situations in frontal, central and parietal regions without signalling hemisphere. Regarding other bands, increased in the frequency band of 8–10 Hz activity was observed within the prefrontal and parietal areas during the Stroop task, and an interaction was assumed between prefrontal and parietal areas [12].

The location of that effect has also been studied using functional neuroimaging, and the results linked selective attention to activity within the dorsolateral prefrontal cortex (DLPFC) and the anterior cingulate cortex (ACC). However, the relative contribution of specific regions involved in the Stroop task remains a continuing source of debate [13]. A number of studies have led to the hypotheses that the left DLPFC may be involved in representing and maintaining the attention demands in this task, while response-related activity is associated with the ACC [14], [15].

Regarding the age effect, there is evidence of age-related increases in interference costs. Indeed, ERP studies showed that the peak latency of the P3 wave was delayed in incongruent trials with respect to congruent ones and that this increase was greater for older rather than younger adults. Comparative studies between young and old populations suggested that age differences in the Stroop interference effect can be explained by a general functional slowing down in the older population, which increases Stroop interference [16], [17].

The purpose of this study was to describe the EEG components involved in Stroop interference, the type of band changes and where they occur within the scalp during the lifetime of individuals. We hypothesized that some bands would remain strong and stable throughout life, while others would show significant changes in older rather than younger subjects. The description of this process should improve our understanding of pathological conditions related to ageing in which attention is severally impaired, such as Parkinson's disease.

## Materials and Methods

### 2.1. Subjects

Ninety healthy volunteers took part in this study, divided into five groups according to each age decade (n_20–29_ = 17, n_30–39_ = 20, n_40–49_ = 17, n_50–59_ = 18, and n_60–69_ = 18). All volunteers were right-handed, as determined by the Edinburgh Handedness Inventory [18] and they had no clinical history of neurological diseases. The University of Murcia ethics committees approved this study. All participants were informed about the aims of the study and the confidential conditions. They also signed an agreement document, in according with the Ethics Committee of the University of Murcia (Spain), where the EEG tests were carried out.

### 2.2. Paradigm

During the EEG tests, subjects were asked to resolve a modified version of the Stroop test [19], as used in previous studies [2] and which involved two kinds of stimuli: 1) incongruent targets, colour names printed in incongruently coloured ink (i.e. *Rojo* [red in English] written in green ink, *Verde* [green in English] written in blue ink, *Azul* [blue in English] written in red ink); and 2) congruent targets, animal names always printed in the same colour (e.g. *Alce* [moose in English] written in blue ink; *Rana* [frog in English] written in red ink; *Visón* [mink in English] written in green ink).

### 2.3. Experimental situation

The experiment was carried out in an electrically-shielded sound-attenuating room. Participants were instructed to answer as soon as possible and to avoid body movement during the recording. Each subject sat on a sofa in the individual sessions, and the stimuli were presented on a plasma TV screen (Samsung LE-32A457, 32 inch, Widescreen, LCD, HD Ready) connected to the main computer and situated 60 cm in front of the sofa. The subject held the experimental keyboard (LUMINA PAD from Cedrus company, model LU430-3B) in his/her right hand and the presentation of the stimuli was carried out using the *Transdatix S.L. software*, which also allows the responses to be recorded (reaction time and correct/incorrect/missing answers). Subjects used a 3-key keyboard: one red, one blue and one green. The stimuli were presented alternatively as 9 trains of 10 congruent stimuli and 9 trains of 10 incongruent stimuli. Each stimulus lasted 3,000 msec, during which time the subject had to reply by pressing the right key. No feedback was provided.

### 2.4. EEG recording

EEGs were recorded continuously using a BrainAmp standard EEG amplifier (256 Hz sampling rate; 0.1–39.9 Hz analogue band pass; resolution 0.5 µv: Brainproducts, Munich, Germany) and a BrainCap with 14 electrodes (Fp_1_, Fp_2_, Fz, C_1_, C_2_, C_3_, C_4_, Cz, T_3_, T_4_, Pz, O_1_ and O_2_) relative to a specific *reference* electrode within the cap between Afz and Fz. The ground electrode was situated between Fz and Cz. A vertical electrooculogram (VEOG) was recorded from electrodes attached above and below the left eye, and the horizontal electrooculogram (HEOG) was obtained from the outer canthi of both eyes (Lansbergen, Kenemans, 2008). The electrode impedance was kept below 5 kΩ, and the EEG and the EOG signals were online band pass filtered (DC-50 Hz, 50 Hz notch filter).

### 2.5. Data Analysis

**Figure 1:**
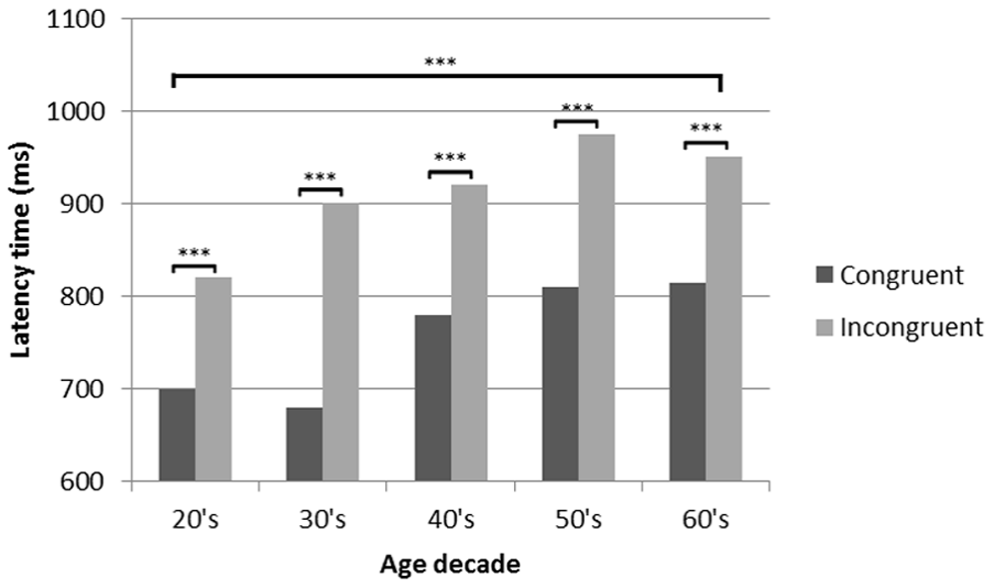
Differences in latency time for congruent and incongruent items per decade. *** stands for *p*<0.001.

Power spectra were computed across the inter-trial interval. EEG time series were divided into non-overlapping 3,000-ms-long windows, beginning at 0 ms post-response. Power spectra were obtained for each window using the Fast Fourier Transform (FFT) by a cosine windowing method. Spectra for each window were averaged separately for congruent and incongruent trials. Statistical analyses were carried out using long-transform mean power values in each frequency band (from 1 to 32 Hz) for the position of all the electrodes. The data from six subjects were rejected due to technical reasons. Repeated-measures ANOVA (rm-ANOVA) included all 14 electrodes as within-subjects factor and group, band and stimuli (congruent, incongruent and resting) as between-subject factors followed by Bonferroni *post hoc* analysis. Furthermore, separate one-way ANOVA was used to assess performance in Stroop test: 1) efficiency was measured as ratio of correct responses (number of correct responses/total number of responses), including colour as a within-subject factor and group as the between-subject factor; 2) reaction time (RT) was evaluated including colour as a within-subject factor and group as the between-subject factor (see Figure 1).

## Results

Rm-ANOVA showed no significant effect of the group factor (F<1). There was significant effect of the within-subject factor band [F(4,890) = 1164.463, p<.001] (Table 1), with the Beta rhythm reaching higher values than the rest of the bands (p<.001), and of the electrodes [F(10,890) = 6.611, p<.002] (Table 2). Conversely, there was no significant effect of “stimulus” as a within-subject factor (F<1). A significant interaction effect on EEG activity was apparent between band and group [F(16,316) = 2.882, p<.004], between electrode and group [F(8,148) = 2.552, p<.015], between band and stimuli [F (4,890) = 1164.463, p<.001] and between stimuli, band and group [F(1252,6320) = 1.252, p<.05]. No further interactions were found.

**Table 1:** Statistical analysis of the EEG Bands per Group (mean, standard deviation and confidence interval).

Bands	Groups	Mean	Std. Error	95% Confidence Interval	
				Lower Bound	Upper Bound
**ALPHA**	1	5.057	0.164	4.735	5.378
	2	5.019	0.153	4.718	5.32
	3	5.133	0.164	4.811	5.454
	4	5.028	0.167	4.7	5.355
	5	4.891	0.173	4.553	5.23
**BETA**	1	9.882	0.164	9.561	10.204
	2	9.144	0.153	8.844	9.445
	3	9.426	0.164	9.105	9.747
	4	10.946	0.167	10.618	11.274
	5	10.856	0.173	10.517	11.194
**DELTA**	1	2.205	0.164	1.883	2.526
	2	2.896	0.153	2.495	3.097
	3	2.163	0.164	1.841	2.484
	4	1.993	0.167	1.665	2.321
	5	2.06	0.173	1.721	2.398
**SUB-ALPHA**	1	3.69	0.164	3.368	4.011
	2	3.595	0.153	3.294	3.896
	3	3.963	0.164	3.642	4.285
	4	3.276	0.167	2.949	3.604
	5	3.267	0.173	2.928	3.606
**THETA**	1	2.46	0.164	2.139	2.781
	2	2.758	0.153	2.457	3.059
	3	2.512	0.164	2.19	2.833
	4	2.078	0.167	1.75	2.406
	5	2.099	0.173	1.761	2.438

**Table 2:** Statistical analysis of the electrodes in function of the stimuli (mean, standard deviation and confidence interval).

Stimuli	electrode	Mean	Std. Error	95% Confidence Interval	
				Lower Bound	Upper Bound
	1	4.598	0.074	4.452	4.743
	2	4.604	0.101	4.405	4.803
	3	4.674	0.075	4.528	4.82
**Incongruent**	4	4.643	0.075	4.497	4.789
**Items**	5	4.659	0.067	4.527	4.89
	6	4.689	0.069	4.543	4.815
	7	4.686	0.074	4.54	4.832
	8	4.659	0.076	4.51	4.809
	9	4.652	0.07	4.515	4.89
	10	4.647	0.074	4.502	4.892
	11	4.692	0.075	4.544	4.84
	1	4.577	0.074	4.431	4.722
	2	4.555	0.101	4.356	4.754
	3	4.668	0.075	4.521	4.814
**Congruent**	4	4.637	0.075	4.491	4.783
**items**	5	4.655	0.067	4.524	4.787
	6	4.682	0.069	4.546	4.818
	7	4.681	0.074	4.535	4.827
	8	4.685	0.076	4.536	4.834
	9	4.666	0.07	4.528	4.804
	10	4.648	0.074	4.503	4.893
	11	4.692	0.075	4.544	4.839
	1	4.665	0.075	4.518	4.812
	2	4.273	0.103	4.072	4.475
	3	4.675	0.075	4.527	4.824
**Resting**	4	4.688	0.075	4.54	4.836
**state**	5	4.699	0.068	4.566	4.832
	6	4.698	0.07	4.56	4.835
	7	4.704	0.075	4.557	4.852
	8	4.652	0.077	4.501	4.803
	9	4.696	0.071	4.557	4.836
	10	4.703	0.075	4.556	4.85
	11	4.718	0.076	4.568	4.867

### 3.1. Band results

The *post hoc* analysis indicated the significant effects indicated below.

#### Alpha Band

##### Congruent stimuli

At electrode Fp2, group 2 showed significantly less Alpha activity than group 5 (t_89_ = −1.944, p<.055).

##### Resting state

At electrodes Fp2, (t_89_ = 2.211, p<.038) and O2 (t_89_ = 1.865, p<.05) group 3 showed significantly stronger Alpha activity than group 5.

#### Beta Band

##### Incongruent stimuli

At electrodes Fp1 (t_89_ = −2.248, p<.027), Fp2 (t_89_ = −1.773, p<.05), C3 (t_89_ = −2.6961.944, p<.009), Cz (t_89_ = −2.405, p<.019), C4 (t_89_ = −3.042, p<.003), T4 (t_89_ = −2.906, p<.005) and O2 (t = −3.707, p<.001) groups 2 and 3 showed significantly weaker Beta activity than group 5.

##### Congruent stimuli

At electrodes Fp1 (t_89_ = −2.835, p<.006), Fp2 (t_89_ = −2.413, p<.018), C3 (t_89_ = −3.129, p<.002), Cz (t_89_ = −3.027, p<.003), C4 (t_89_ = −2.618, p<.011), T4 (t_89_ = −2.339, p<.022) and O2 (t_89_ = −3.055, p<.003) groups 2 and 3 showed significantly weaker Beta activity than group 5.

##### Resting state

At the electrode Fp1 (t_89_ = −2.258, p<.027), Fp2 (t_89_ = −2.244, p<.028), C4 (t_89_ = −2.656, p<.010), T4 (t_89_ = −2.656, p<.010) and O2 (t_89_ = −2.099, p<.039) group 2 showed significantly weaker Beta activity than group 5. Furthermore, at Fp2, group 4 displayed significantly weaker Beta activity than group 5.

#### Delta Band

##### Incongruent stimuli

At electrode Pz (t_89_ = 1.988, p<05) group 2 showed significantly stronger Delta activity than group 5. Furthermore, at electrode Fp2, group 1 (t_89_ = 2.238, p<.05), group 2 (t_89_ = 3.277, p<.002), group 3 (t_89_ = 1.942, p<.05) and group 4 (t_89_ = 1.900 p<.05) displayed significantly stronger Delta activity than group 5.

##### Congruent stimuli

At the Fp2 electrodes (t_89_ = 3.440, p<.028), T3 (t_89_ = 2.068, p<.042), the T4 (t_89_ = 2.150, p<.035) and O2 (t_89_ = 2.609, p<.011) groups 2 showed significantly stronger Delta activity than group 5. Furthermore, at electrode Fp2. group 1 (t_89_ = 2.238, p<.028), group 3 (t_89_ = 2.596, p<.011) and group 4 (t_89_ = −2.996 p<.004) displayed significantly stronger Delta activity than group 5.

##### Resting state

Within electrode Fp1 (t_89_ = −2.377, p<.02) group 4 showed significantly less Delta activity than group 5. At the Fp2 electrode (t_89_ = 2.370, p<.02) group 1 showed significantly stronger Delta activity than group 5. Furthermore, at electrode T3 (t_89_ = 3.58, p<.003), C4 (t_89_ = 2.526, p<.014), Cz (t_89_ = 2.012, p<.048), C4 (t_89_ = 2.703, p<.008), T4 (t_89_ = 2.274, p<.025) and Pz (t_89_ = 2.546, p<.013) group 2 showed significantly stronger Delta activity than group 5.

#### Sub-Alpha Band

##### Incongruent stimuli

At electrode C3 (t_89_ = 3.580, p<.003), Cz (t_89_ = 2.767, p<.007), Cz (t_89_ = 2.587, p<.012) and C4 (t_89_ = 2.200, p<.029), group 3 showed significantly stronger Sub-Alpha activity than group 5.

##### Congruent stimuli

At the Fp1 (t_89_ = 2.316, p<.023) and Fpz (t_89_ = 2.473, p<.016) electrodes, group 1 showed significantly stronger Sub-Alpha activity than group 5. Furthermore, at Fpz, group 3 (t_89_ = 2.955, p<.004) displayed significantly stronger Sub-Alpha activity than group 5.

##### Resting state

At electrode Fp1, group 1 (t_89_ = 2.248, p<.027) and group 2 (t_89_ = 2.009, p<.048) showed significantly stronger Sub-Alpha activity than group 5.

#### Theta Band

##### Incongruent stimuli

At electrodes Fp1 (t_89_ = 1.758, p<.05), T3 (t_89_ = 2.843, p<.006), C3 (t_89_ = 3.142, p<.002), Cz (t_89_ = 1.950, p<.05), C4 (t_89_ = 2.536, p<.013), T4 (t_89_ = 2.301, p<.024) and O2 (t_89_ = 2.276, p<.026), group 2 showed significantly stronger Theta activity than group 5. Furthermore, at electrode Fp2, group 3 achieved stronger Theta activity than group 5 (t_89_ = 2.613, p<.05).

##### Congruent stimuli

At electrodes Fp2 (t_89_ = 2.613, p<.011), C3 (t_89_ = 2.401, p<.019), C4 (t_89_ = 1.91, p<.05), groups 2 and 3 showed significantly stronger Theta activity than group 5. Furthermore, at electrode C3, group 1 displayed significantly stronger Theta activity than group 5 (t_89_ = 1.980, p<05).

##### Resting State

Among the Fp2 (t_89_ = 3.166, p<.002), T3 (t_89_ = 2.974, p<.004), C3 (t_89_ = 1.842, p<.05), Cz (t_89_ = 4.049, p<.044), C4 (t_89_ = 3.674, p<.001), T4 (t_89_ = 1.866, p<.05) and Pz (t_89_ = 2.627, p<.01) electrodes, group 2 showed significantly stronger Theta activity than group 5. Furthermore, at electrode Fp2 (t_89_ = 2.774, p<.007) and C3 (t_89_ = 2.451, p<.016) group 3 displayed stronger Theta activity than group 5. Finally, at electrode T3 (t_89_ = 2.084, p<.04) the Theta activity in group 1 was enhanced with respect to group 5.

### 3.2 Stroop test results

The conflict effect in this Stroop task was verified for each age group (p<.001, Figure 1). In terms of the ratio of correct responses, rm-ANOVA showed a significant effect of the group factor [F(4,53) = 12.258, p<.001] but no effect of colour (p>.005). A *Post hoc* analysis indicated significant differences in groups 1, 2, 3 and 4 with respect to group 5 (p>.001). With regards reaction time, there was a significant effect of the group factor [F(16,890) = 5.985, p<.001], the *post hoc* t-test analysis indicating significant differences of groups 1 (t_89_ = 6.332, p<.001), 2 (t_89_ = 6.334, p<.001), 3 (t_89_ = 6.298, p<.001) and 4 (t_89_ = 5.813, p<.001) with respect to group 5.

## Discussion

In this study we have analysed the changes in Stroop interference at different stages in the life of individuals, analysing the responses within specific bands at electrodes placed at different locations during Stroop task performance. Our results suggest that a complex combination of changes in the Alpha and Theta bands evolve between ages in the 20's until the 70's together with a progressive increase in latency of response between congruent and incongruent items.

In general terms, alertness is characterized by reduced Alpha activity and increases in the rest of the frequencies [20]. In particular, increase in Theta activity is related to information processing and contributes to cognitive function such as memory encoding engagement [21], [22], learning [23] and creativity processing [24]. During conflict resolution, the reduction in Alpha activity corresponded to diffuse electrical inhibition over the scalp that was required to resolve any demanding cognitive tasks. This process is essential to guarantee a correct analysis of the information and correct processing, mainly within parietal areas. In our study, conflict solving produced a reduction of Alpha waves in the right occipital lobe (O2), particularly in older groups (those in their 60's) and an increase in the Theta frequency at Fp2. The location O2 corresponds to the parietal homotypical isocortex from the parieto-occipital region, which is involved in resuming and processing visual information. Simultaneously, Theta activity increased within the prefrontal lobes (Fp1 and Fp2), frontal lobes (F3 and C3), frontal sagittal line (Fz) and central sagittal line (Cz), being higher at Fp2. The Right Frontopolar cortex (Fp2) is essential for the processing of information received from the associative cortex, and is in continuous exchange with memory areas [25]. Petrides [26] described the implication of prefrontal areas during the Stroop test: the anterior fronto-basal region (area 11) is involved in novelty flagging (this requires memory connexions, [27]) and the posterior fronto-basal region (area 13) contributes to the meaning analysis of the stimulus; in case of incongruence, signal analysis would require the activation of areas 11 and 13, which are connected to area 25 (Subgenual), the amygdala entorhinal and the perirhinal cortex for meaning elaboration [28]. The peak of Theta activity under Fp2 resembles the confluence of lateral prefrontal and anterior fronto-basal electrical fields, which respond to data comparison and memory processing. Theta activity increase was also found under electrode Fz, which corresponds to the confluence of area 13 (posterior fronto-basal cortex) and areas 32a and 24 (anterior pregenual cortex, limbic system) electrical fields. Cz electrode activity corresponds to areas 32b and 24b (anterior cingulate cortex), as described for both congruent and incongruent items using fMRI and PET techniques [2], [29], [30]. Further areas, such as the left premotor area (F3) and the left motor area (C3), would stand for the motor response, performed by pressing a button with the right hand. Such a complex and long process, particularly in incongruent analysis, originates longer reaction time and more errors, leading to the so-called “Stroop effect”.

These results are in agreement with the relationships previously identified between central executive, working memory processes and fronto-parietal electrode coupling [31]. Moreover, the general functional scheme used here matches that applied in a previous study where similar relationships between central executive and working memory processes, and fronto-parietal electrode coupling were described [32]. Regarding the specific location of the changes in EEG signal, our results confirm that Stroop interference involves the right frontal cortex (lateral and basal prefrontal areas –Fp2-), and posterior fronto-sagittal ones, Fz, as proposed from previous fMRI clinical studies in healthy controls and patients with schizophrenia [33].

Considering the whole pool of data by decades across the sample and irrespective of age, reaction time was significantly shorter for congruent than for incongruent items. Regarding the effect of age in relation with the Stroop incongruence, our results indicated that older adults have a longer reaction time for both congruent and incongruent items. However, response time was significantly shorter for the younger participants than for the older participants on congruent and incongruent items (younger: 700 ms for congruent and 825 ms for incongruent; older: 725 ms for congruent and 1250 ms for incongruent). These data suggest an increase of 25 ms/decade for congruent and 85 ms/decade for incongruent items, probably due to the contribution of different aging processes that may start from age 20. These is in agreement with previous studies: the Stroop test in different age groups reported decreases in reaction times to incongruent stimuli from 30 to 20 years of age (−0.5 z scores) and these times start to increase from 40 years of age onwards, at a rate of 0.2 z-scores/decade [34].

Consistent with well-described anatomical changes, Stroop interference reaches adaptive levels relatively early in childhood (6–7 years), although control interference continues to develop into late adolescence [35]. In fact, 10–12 year-old subjects are still more susceptible to interference errors than adults [36]. Likewise, previous studies revealed age-related differences within Correct Response Negativity (CRN) amplitude and CRN amplitude was larger after incongruent than congruent Stroop stimuli in young adults, whereas older adults showed greater amplitude of CRN in both incompatible as well as compatible trials. Hence, there appeared to be an age-related impairment in (post-)response conflict [37]–[39]. These effects are connected with other age-related anatomical changes in the brain associated with age, such as the increased of ventricle volume (10–15%) from the 40^th^ to the 80^th^ decade in the healthy population [40]. Standard ageing processes may start age 20 [24], although some ageing parameters such as myelinisation increase through age 40 and in some cases until age 60, especially in the intracortical horizontal plexuses [41]. Within the central nervous system, standard ageing changes include microcirculation decrease [42], [43], ventricles enlargement [44], white matter reduction [45]–[47] and encephalic weight loss [48]. Slowing in responses with age may be well attributed to standard ageing changes in the central nervous system.

In summary, our results highlight the cortical areas involved in conflict resolution, implicating the right posterior parietal or occipito-parietal, the fronto-basal and the left ACC. The EEG frequency that best defines the engagement of these areas is represented by the appearance of 4–6 Hz Theta activity in Fp2 (some peaks of which may reach 9 Hz), and the simultaneous reduction of Alpha and Beta rhythms. The brain's ability to swap these EEG activity bands is crucial to achieve efficient performance [31]. Such a key combination seems not to be optimal in the 20's but rather in the 30's, and from then on this starts to decline until the 60^th^ decade as healthy aging occurs. We reckon that our results enhance our current knowledge on the EEG changes that take place under cognitive demanding conditions during the life span of an individual. However, further studies should consider to increase the number of electrodes [49] and apply basal interpolation software to identify the contribution of each EEG component. These data are being used in the EXOLEGS project, which aims to improve the capacity of autonomy of elderly and impaired people by the application of user interface techniques, focusing mainly in the chapter of Brain Computer Interface.
